# Enzymatic Oxidation of Cholesterol: Properties and Functional Effects of Cholestenone in Cell Membranes

**DOI:** 10.1371/journal.pone.0103743

**Published:** 2014-08-26

**Authors:** Maarit Neuvonen, Moutusi Manna, Sini Mokkila, Matti Javanainen, Tomasz Rog, Zheng Liu, Robert Bittman, Ilpo Vattulainen, Elina Ikonen

**Affiliations:** 1 Institute of Biomedicine, Anatomy, University of Helsinki, Helsinki, Finland; 2 Department of Physics, Tampere University of Technology, Tampere, Finland; 3 Department of Chemistry and Biochemistry, Queens College, The City University of New York, Flushing, NY, United States of America; 4 MEMPHYS – Center of Biomembrane Physics, University of Southern Denmark, Odense, Denmark; 5 Minerva Foundation Institute for Medical Research, Helsinki, Finland; University of Copenhagen, Denmark

## Abstract

Bacterial cholesterol oxidase is commonly used as an experimental tool to reduce cellular cholesterol content. That the treatment also generates the poorly degradable metabolite 4-cholesten-3-one (cholestenone) has received less attention. Here, we investigated the membrane partitioning of cholestenone using simulations and cell biological experiments and assessed the functional effects of cholestenone in human cells. Atomistic simulations predicted that cholestenone reduces membrane order, undergoes faster flip-flop and desorbs more readily from membranes than cholesterol. In primary human fibroblasts, cholestenone was released from membranes to physiological extracellular acceptors more avidly than cholesterol, but without acceptors it remained in cells over a day. To address the functional effects of cholestenone, we studied fibroblast migration during wound healing. When cells were either cholesterol oxidase treated or part of cellular cholesterol was exchanged for cholestenone with cyclodextrin, cell migration during 22 h was markedly inhibited. Instead, when a similar fraction of cholesterol was removed using cyclodextrin, cells replenished their cholesterol content in 3 h and migrated similarly to control cells. Thus, cholesterol oxidation produces long-term functional effects in cells and these are in part due to the generated membrane active cholestenone.

## Introduction

Cholesterol is a vital constituent in the plasma membrane of higher eukaryotes, where it typically represents 25–40% of total lipids [1], [2]. Cholesterol regulates biophysical membrane properties such as fluidity, permeability, and rigidity. It interacts with neighbouring lipids and proteins via steric interactions and via hydrogen bonding through its 3β-hydroxyl group. The interactions between cholesterol and polar phospholipids can locally increase lipid order. This leads to the formation of dynamic membrane domains that contribute to the regulation of key cellular processes, such as receptor signaling, endocytosis and cell polarity [3], [4].

To assess the functional importance of cholesterol, membrane cholesterol content is often reduced experimentally. Typically, cholesterol is extracted using methyl-β-cyclodextrin (MBCD), which can deplete up to 80–90% of plasma membrane cholesterol [5]. Another commonly used method is to expose the membrane to purified bacterial cholesterol oxidase (coase) [4], [6]–[10]. Enzymatic cholesterol oxidation and cholesterol removal by MBCD are often used interchangeably for cholesterol reduction but they act via different mechanisms; MBCD extrudes cholesterol from the membrane, whereas coase catalyzes the conversion of up to ∼20% of cellular cholesterol to 4-cholesten-3-one (cholestenone) [7], [11], [12]. Cholesterol oxidizing bacteria can further catabolize cholestenone to use it as their nutritional hydrocarbon source. However, in mammals, cholestenone is metabolized primarily in the liver [13], [14]. Therefore, once generated, cholestenone is likely to persist in extrahepatic mammalian cells.

In cholestenone, the steroid 3-hydroxyl group is replaced by a keto group, with a more limited capacity for hydrogen bonding than a hydroxyl group. Consequently, cholestenone preferentially localizes to liquid-disordered (L_d_) domains in model membranes and causes lipid monolayer expansion [12], [15], [16]. While coase treatment is widely used to disturb cholesterol domains in cell membranes [4], [7], [10], [17], [18], the membrane partitioning and effects of cholestenone in the cellular context have so far received little attention.

In this study, we characterized the effects of coase treatment on membrane order and steroid mobility in primary human dermal fibroblasts (HDFs). The molecular interactions involved in cholestenone membrane partitioning and desorption from the membrane were addressed using atomistic simulations. Our data suggest that cholestenone is highly mobile in membranes and influences cholesterol flip-flop and efflux. Moreover, we provide evidence that in contrast to MBCD induced cholesterol depletion cholesterol oxidation causes long-term functional defects in cells due to the persistence of cholestenone.

## Results and Discussion

### Cholesterol oxidation reduces membrane order and increases steroid flip-flop

**Figure 1:**
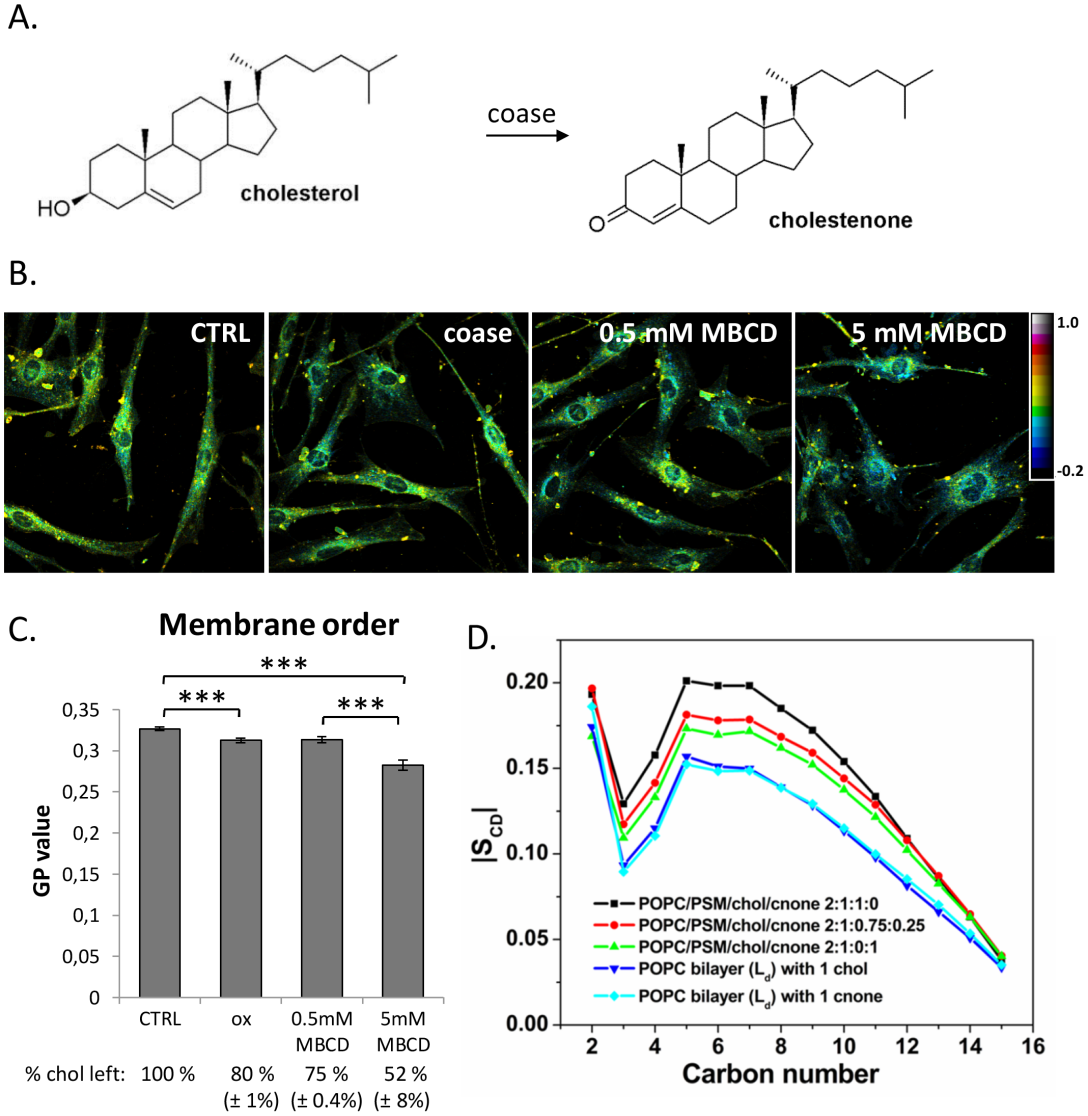
Cholesterol oxidation decreases membrane order. A) Structures of cholesterol and cholestenone. B) HDFs were treated at 37°C with coase (10 U/ml; 1 h), MBCD (0.5 mM; 5 min or 5 mM; 1 h) or buffer only (ctrl). Cells were labeled with Laurdan, fixed, imaged with a 2-photon microscope and GP images were generated. C) Average GP values calculated from the Laurdan GP data represent three independent experiments; n = 59 fields, >300 cells per condition; ***P<0.0005. The fraction of cholesterol remaining in cells after the treatments is indicated. D) The order parameters |S_CD_|, from MD simulations of the palmitoyl tail of POPC molecules in various lipid bilayers. The liquid-ordered (L_o_) systems were POPC/PSM/cholesterol/cholestenone 2∶1∶1∶0, POPC/PSM/cholesterol/cholestenone 2∶1∶0.75∶0.25, POPC/PSM/cholesterol/cholestenone 2∶1∶0∶1, and the liquid-disordered (L_d_) systems were with 1 chol in a POPC bilayer and 1 cholestenone in a POPC bilayer. Carbon numbering starts from carbons close to the glycerol group.

To assess if coase treatment (**Figure 1A**) affects membrane fluidity in HDFs, we analysed the generalized polarization (GP) of the fluorescent probe Laurdan. Coase treatment at 37°C for 1 h converted approximately 20% of cellular cholesterol into cholestenone. This treatment was compared to moderate or severe cholesterol depletion by MBCD, extracting ∼25% or ∼50% of cellular cholesterol, respectively. We found that cholesterol oxidation and moderate MBCD-mediated cholesterol depletion resulted in a similar degree of GP reduction, while severe cholesterol depletion caused a larger decrease in GP (**Figure 1B, C**). Thus, the range of increase in membrane fluidity was roughly proportional to the level of cholesterol depletion (R^2^ = 0.95). However, it is worth noting that MBCD treatment may also result in the extraction of other membrane lipids [5].

In line with GP measurements, atomistic molecular dynamics (MD) simulations in a raft-like bilayer (palmitoyl-oleyl phosphatidylcholine (POPC): N-palmitoyl-sphingomyelin (PSM): cholesterol, molar ratio 2∶1∶1) predicted a substantial decrease in the ordering of the palmitoyl chain of POPC when 25% of cholesterol was replaced by cholestenone (**Figure 1D**). Interestingly, 25% replacement of cholesterol by cholestenone decreased palmitoyl chain ordering almost as much as complete replacement of cholesterol by cholestenone, indicating that cholestenone affects the ordering capability of the remaining cholesterol. However, all of the simulated raft-like membranes with a total of 25 mol% steroid were more ordered than a liquid-disordered bilayer (**Figure 1D**). In addition, the replacement of cholesterol by cholestenone had a minor effect on the lateral diffusion of PSM, increasing it at short times (**Figure S1**).

**Figure 2:**
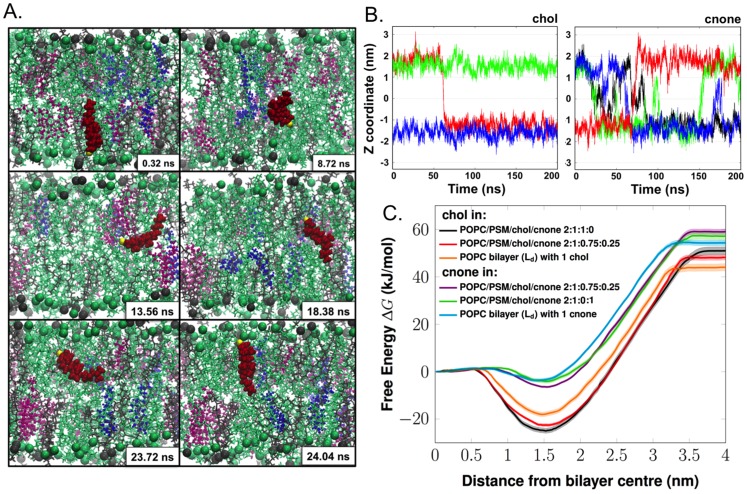
Transbilayer mobility of cholestenone and cholesterol. A) Snapshots showing the spontaneous flip-flop motion of a cholestenone molecule during MD simulation in the raft-like system POPC/PSM/cholesterol/cholestenone 2∶1∶0.75∶0.25. POPC and PSM are shown as green and grey lines, respectively, with head-group phosphorus atoms as spheres. Cholesterol (chol) and cholestenone (cnone) are shown as magenta and blue ball-and-stick representation, respectively. The particular cholestenone undergoing flip-flop is highlighted as red van der Waals spheres and its 3-keto oxygen atom is shown in yellow. B) Time scale and frequency of flip-flops by cholesterol and cholestenone. Time evolution of the Z-coordinate of the head group oxygen atom of cholesterol (i) and cholestenone (ii) along the bilayer normal in the system POPC/PSM/chol/cholestenone 2∶1∶0.8∶0.2. The Y-axis zero indicates the center of the bilayer. Different colors show different cholesterol or cholestenone molecules. The figures highlight how different the flip-flop rates of cholesterol and cholestenone are: while cholesterol undergoes only a single quick flip-flop event, cholestenone undergoes a large number of flip-flops during the same period. Yet the concentration of cholesterol was four times higher than that of cholestenone. C) The potential of mean forces (kJ/mol) of cholesterol and cholestenone in a variety of lipid bilayer models (shown by dark lines), either in the liquid-disordered (L_d_) or liquid-ordered (L_o_) phase. The transparent area following each PMF curve represents the corresponding statistical errors.

Atomistic MD simulations were also used to analyse how cholestenone affects the movement of the steroid molecules between membrane leaflets. One of the most striking observations was the frequent occurrence of inter-leaflet movements or flip-flop motions of cholestenone molecules (**Figure 2A, B**). In contrast to cholestenone, cholesterol exhibited a very stable vertical position along the bilayer normal, with rare flip-flops (**Figure 2B**). The increased flip-flop rate of cholestenone is in accordance with its decreased hydrogen-bonding capacity compared with that of cholesterol (**Table 1**).

**Table 1:** Average number of hydrogen bonds^a^ (H-bonds) and charge pairs^b^ formed by steroids with the surrounding lipids and water.

System	H-bonds with lipids		H-bonds with water		Charge pairs	
	chol	cnone	chol	cnone	chol	cnone
**I_MD_** (POPC/PSM/chol/cnone 2∶1∶1∶0)	0.37 (0.17/0.20)*	-	1.66	-	0.61	-
**II_MD_** (POPC/PSM/chol/cnone 2∶1∶0.8∶0.2)	0.35 (0.18/017)*	0.04	1.67	0.53	0.60	0.46

*(per POPC, or per PSM).

a The presence of H-bonds was evaluated based on distance and angle criteria: the donor- acceptor distance had to be less than or equal to 0.325 nm, and the angle between the vector linking a donor and an acceptor, and the vector describing the chemical bond between hydrogen and a donor had to be less than or equal to 35°.

b The existence of charge pairs was determined using the distance cut-off of 0.4 nm.

Errors are less than 0.01.

To gain a more quantitative view of the steroid inter-leaflet movements, we performed free energy calculations using umbrella sampling [19]. The free energy profiles are shown in **Figure 2C**, and the free energy values associated with these processes in **Table 2**. The results obtained for cholesterol are in agreement with previous studies [20]–[22]. The free energy of both steroids had a minimum at the equilibrium position (with oxygen located at the bilayer interface) and increased toward the bilayer center as well as toward bulk water (**Figure 2C**).

**Table 2:** Thermodynamics and kinetics associated with cholesterol/cholestenone flip-flop and desorption.

Model system		ΔG_desorb_ a (kJ/mol)	ΔG_barrier_ b (kJ/mol)	ΔG_center_ c (kJ/mol)	t_d_ (ns)	k_d_ (S^−1^)	k_f_ (S^−1^)	k_flip_ (S^−1^)
**chol**	I_FE_ (POPC/PSM/chol/cnone 2∶1∶1∶0)	76.0±2.7	26.0±1.2	25.0±1.0	1–80	1.3×10^7^ to 10^9^	7.8×10^2^ to 6.0×10^4^	4.0×10^2^ to 3×10^4^
	II_FE_ (POPC/PSM/chol/cnone 2∶1∶0.75∶0.25)	70.6±1.6	23.5±0.9	22.5±0.6	1–80	1.3×10^7^ to 10^9^	2.2×10^3^ to 1.7×10^5^	1.1×10^3^ to 0.9×10^5^
	IV_FE_ (127 POPCs and 1 chol)	62±2.4	19.0±1.3	18.0±0.9	1–80	1.3×10^7^ to 10^9^	1.2×10^4^ to 9.1×10^5^	0.6×10^4^ to 4.6×10^5^
**cnone**	II_FE_ (POPC/PSM/chol/cnone 2∶1∶0.75∶0.25)	65.4±1.5	8.0±0.6	6.4±0.3	1–40	2.5×10^7^ to 10^9^	2.1×10^6^ to 8.2×10^7^	1.1×10^6^ to 4.1×10^7^
	III_FE_ (POPC/PSM/chol/cnone 2∶1∶0∶1)	61.3±2.3	5.8±0.9	4.1±0.7	1–40	2.5×10^7^ to 10^9^	5.0×10^6^ to 2.0×10^8^	2.5×10^6^ to 10^8^
	V_FE_ (127 POPCs and 1 cnone)	57.8±1.7	4.9±0.9	3.5±0.6	1–40	2.5×10^7^ to 10^9^	6.3×10^6^ to 2.5×10^8^	3.2×10^6^ to 1.3×10^8^

a ΔG_desorb_ is the free energy barrier for desorption from membrane to water phase.

b ΔG_barrier_ is the free energy barrier for flip-flop.

c ΔG_center_ is the difference in free energy between the bilayer center (Z_center_) and the equilibrium position (Z_eq_).

The parameters k_d_, k_f_, k_flip_, and t_d_ are defined and discussed in Materials and Methods.

The difference in the flip-flop movements of cholesterol and cholestenone was reflected in their free-energy values. We found that the free energy barrier for cholestenone flip-flop was about four times lower than that of cholesterol (**Figure 2C** **,** **Table 2**). Consequently, cholestenone flip-flop took place on a nanosecond timescale, whereas cholesterol flip-flop occurred on a timescale of microseconds to milliseconds (**Table 2**). Also, the flip-flop rate of cholesterol was increased 3-fold in the presence of cholestenone, whereas cholestenone flip-flop was consistently fast and less dependent on other membrane lipids (**Table 2**). Increased flip-flop is likely to decrease the lateral pressure and increase elasticity of the membrane [23], [24]. No pore formation was observed, unlike in flip-flops of phospholipids [25], [26].

Atomistic simulations predicted that cholestenone desorbs from the membrane to the water phase more readily than cholesterol. In a raft-like membrane containing a mixture of cholesterol and cholestenone, the free energy barriers for desorption were 70.6 and 65.4 kJ/mol for cholesterol and cholestenone, respectively. In a L_d_ membrane, the corresponding values were 62 and 57.8 kJ/mol. The simulations thereby showed a consistent 7.3% reduction in the free energy barrier for cholestenone compared with cholesterol, suggesting that cholestenone fluxes from the membrane more easily than cholesterol.

### Cholestenone is transferred from HDFs to extracellular acceptors more readily than cholesterol

**Figure 3:**
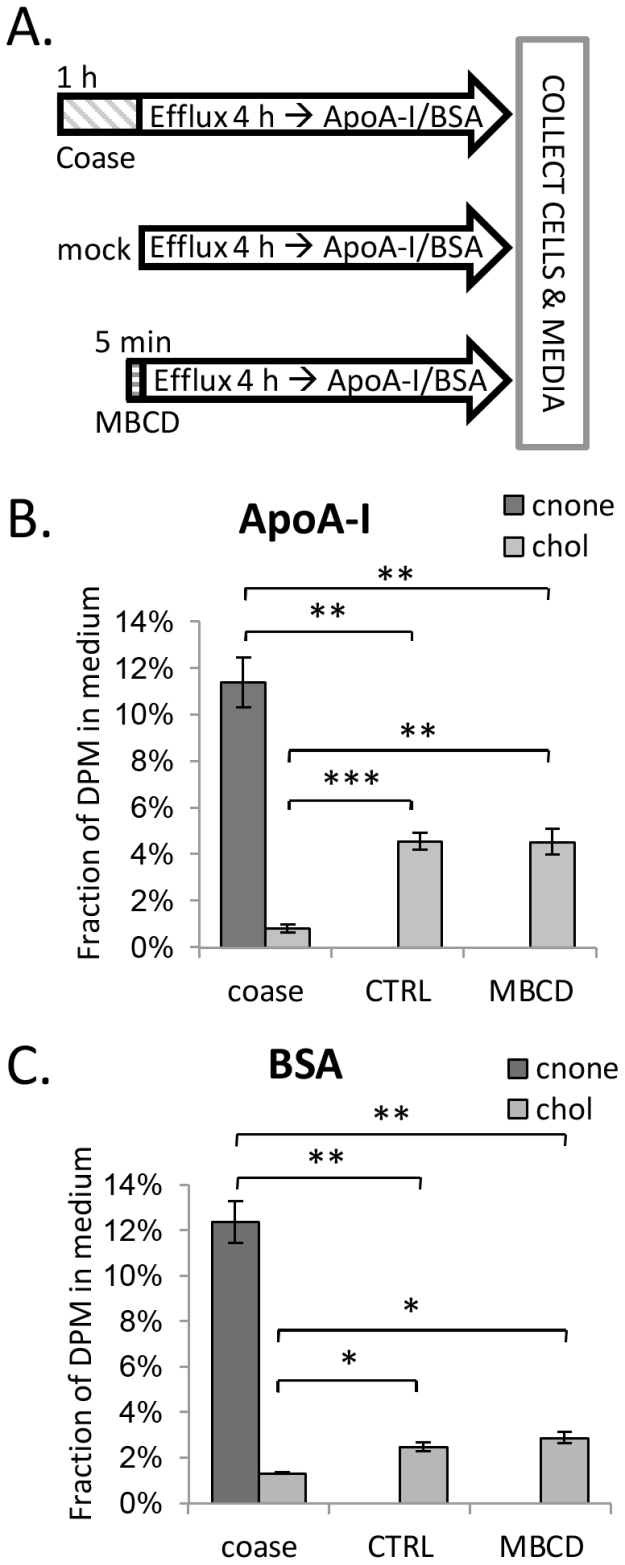
Efflux of cholestenone and cholesterol from HDFs to extracellular acceptors. A) HDFs prelabeled with [^14^C]cholesterol were treated with coase (10 U/ml; 1 h), MBCD (0.5 mM; 5 min), or mock treated (ctrl). Efflux of radiolabeled lipids was analyzed after a 4 h chase in serum-free buffer containing B) ApoA-I (10 µg/ml); n = 4 or C) BSA (0.2%); n = 4–6. *P<0.05; **P<0.005; ***P<0.0005.

To study if cholestenone is released more avidly than cholesterol from biological membranes, we analysed the efflux of radiolabeled cholesterol and cholestenone from HDFs. For this purpose, HDFs were labeled with [^14^C]cholesterol for 42 h, and then oxidized with coase (10 U/ml) for 1 h to produce [^14^C]cholestenone. This resulted in the oxidation of 17.4±2.7% of [^14^C]cholesterol. In control experiments, cells were either mock treated or treated with 0.5 mM MBCD to deplete ∼25% of [^14^C]cholesterol. The efflux of radiolabeled cholesterol and cholestenone from HDFs was analysed after 4 h of chase in a buffer containing either Apolipoprotein A-I (ApoA-I) or bovine serum albumin (BSA) as lipid acceptors (**Figure 3A**).

We observed that [^14^C]cholestenone underwent efflux from HDFs much more efficiently than [^14^C]cholesterol: the efflux of [^14^C]cholestenone to ApoA-I was ∼2.5 times higher than that of [^14^C]cholesterol from control cells (**Figure 3B**). The difference was even more pronounced when BSA was used as the acceptor, with the proportion of [^14^C]cholestenone delivered to BSA being roughly 5 times higher than that of [^14^C]cholesterol in control cells (**Figure 3C**). Interestingly, the efflux of [^14^C]cholesterol to ApoA-I or BSA was markedly reduced in coase-treated cells, while MBCD pretreatment did not affect this: Approximately 5% of [^14^C]cholesterol was released to ApoA-I and ∼2% to BSA in control and MBCD-treated cells; instead, when cells were pretreated with coase, only 1% of [^14^C]cholesterol was transferred to ApoA-I or BSA (**Figure 3B, C**).

Thus, cholestenone was more readily transferred from HDF membranes to extracellular acceptors than cholesterol. Moreover, introduction of cholestenone into the membrane inhibited the release of cholesterol to extracellular acceptors. This is in apparent contradiction to our simulation results where the presence of cholestenone rather facilitated cholesterol desorption (**Table 2**). The reason for this difference is not clear, but one possibility is that the reduced cholesterol efflux in coase treated cells results from oxidation of cholesterol in domains that are only slowly replenished via protein dependent pathway(s). For instance, the key cholesterol efflux protein ABCA1 may play a role in transferring cholesterol to less packed domains susceptible to oxidation. This would be in line with the finding that ABCA1 activity increases cholestenone production by coase [27], [28].

### Partitioning of cholestenone and BODIPY-cholestenone in cellular membranes

**Figure 4:**
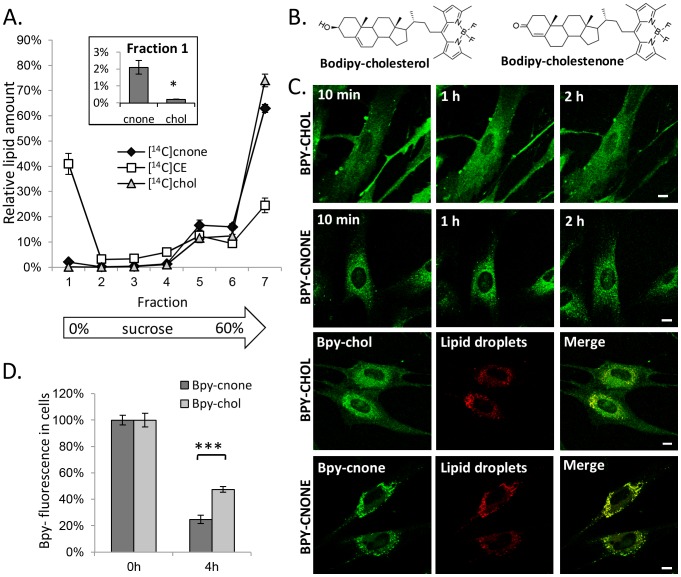
Intracellular trafficking of cholesterol and cholestenone. A) RAW 264.7 macrophages prelabeled with [^14^C]cholesterol for 18 h were treated with 10 U/ml coase for 2 h. The post-nuclear supernatant was subjected to sucrose gradient fractionation. A total of 7 fractions was collected and analysed for radiolabeled [^14^C]cholesterol, -cholestenone, and -cholesteryl ester content. Inset: Percentage of [^14^C]cholesterol and -cholestenone in the lowest density fraction 1; n = 4. *P<0.05. B) Structures of Bpy-cholesterol (Bpy-chol) and Bpy-cholestenone (Bpy-cnone). C) HDFs were pulse labeled with the indicated Bpy-lipids for 8 min, and the cells were imaged by confocal microscopy during 2 h of chase. To visualize LDs, cells were pulse labeled for 1 h with Bpy568-C12 (5 µM) and Bpy-lipid (1 µM), and imaged after a 1 h chase. Scale bars 10 µm. D) HDFs were pulse labeled with Bpy-lipids and analysed for Bpy fluorescence intensity immediately after the pulse and after 4 h of chase in DMEM, 10% FBS; n = 8; ***P<0.0005.

We next analysed the partitioning of [^14^C]cholesterol and [^14^C]cholestenone (generated by coase) in cellular membrane fractions using sucrose gradient centrifugation. This showed that the distributions of [^14^C]cholesterol and [^14^C]cholestenone were similar apart from the slight preference of [^14^C]cholestenone for lower density fractions, as evidenced by its relative enrichment in the top fraction containing lipid droplets (LDs, enriched in [^14^C]cholesteryl esters) and depletion in the bottom fraction, as compared to [^14^C]cholesterol (**Figure 4A**).

To visualize how the removal of cholesterol's 3β-hydroxyl group affects lipid mobility in cell membranes, we synthesized a fluorescently labeled analogue, BODIPY-cholestenone (Bpy-cholestenone) (**Figure 4B**). In this lipid, the hydroxyl group of the earlier characterized sterol analogue BODIPY-cholesterol (Bpy-cholesterol; [29]) is replaced by a keto group, and the position of the 5,6-double bond is changed to a 4,5-double bond, as in native cholestenone. The trafficking of Bpy-cholesterol and Bpy-cholestenone was compared in living HDFs upon labeling from MBCD-Bpy-lipid complexes. Bpy-cholesterol was localized mainly in the plasma membrane directly after labeling (**Figure 4C**) and from there, transported to intracellular compartments as described [29], [30], becoming concentrated in the perinuclear area at 2 h post labeling (**Figure 4C**). In contrast, the vast majority of Bpy-cholestenone was observed in intracellular compartments immediately after labeling and remained in these structures (**Figure 4C**). This compartment was identified as LDs by co-labeling with BODIPY558/568-C12 (**Figure 4C**). Bpy-cholesterol also colocalized with LDs to some extent, but less prominently than Bpy-cholestenone. These data indicate that the 3β-hydroxyl group is critical for the intracellular trafficking of the Bpy-sterol. The prominent LD localization of Bpy-cholestenone compared to the modest LD affinity of [^14^C]cholestenone suggests that the hydrophobic Bpy-moiety largely drives the LD localization of the fluorescent derivative lacking the 3β-hydroxyl group.

We also studied the removal of the Bpy-lipids from HDFs to extracellular acceptors. At 4 h of incubation in serum-containing medium, ∼50% of Bpy-cholesterol fluorescence was retained in the cells, whereas only ∼25% of Bpy-cholestenone was retained (**Figure 4D**). This shows that Bpy-cholestenone was more efficiently effluxed from cells than Bpy-cholesterol, analogously to the radiolabeled sterols.

### Cholestenone generated upon cholesterol oxidation restrains cell migration

**Figure 5:**
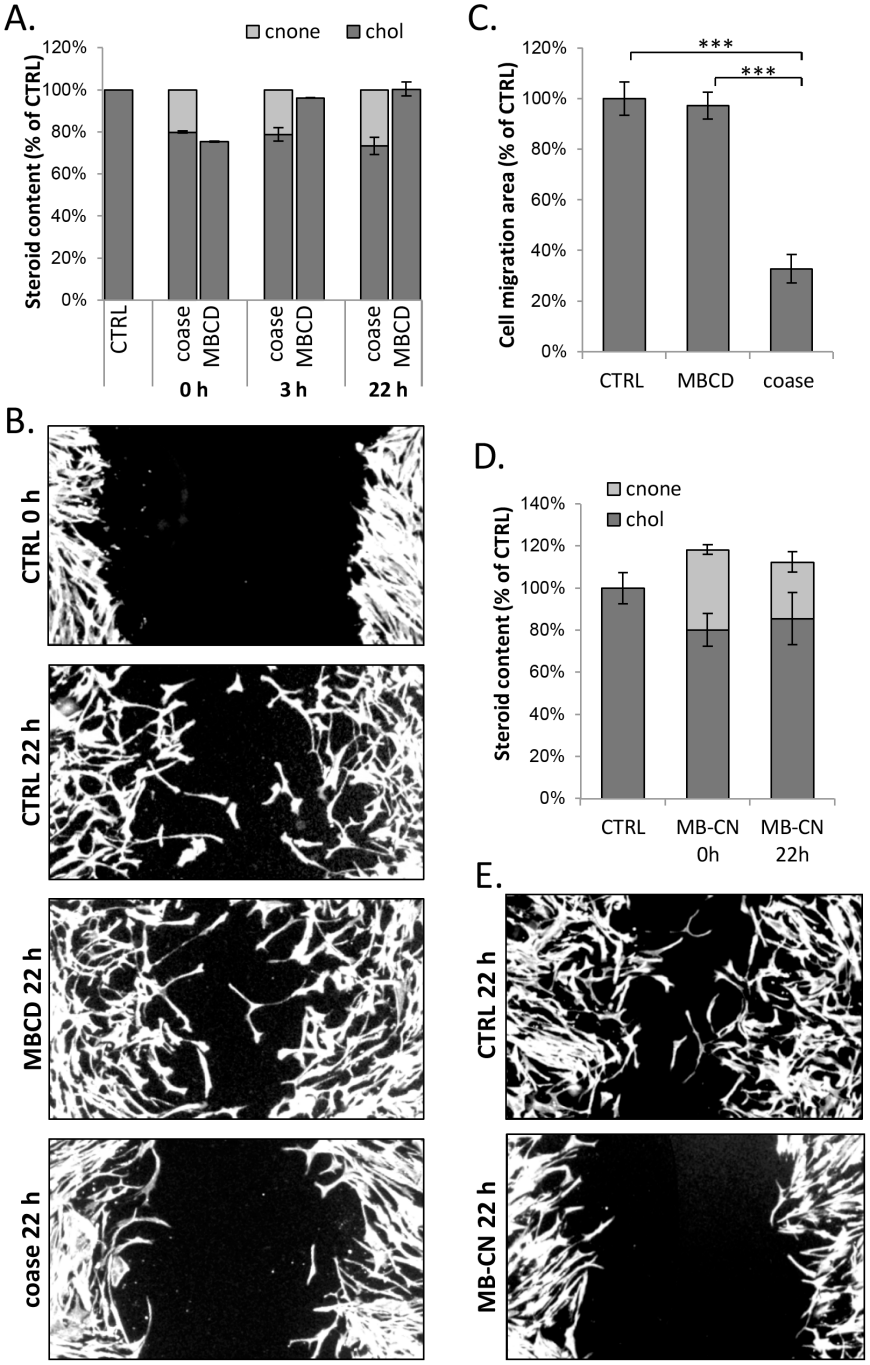
Coase treatment or cholestenone impairs fibroblast migration in a wound-healing assay. A) Confluent HDFs were treated with coase (10 U/ml; 1 h), MBCD (0.5 mM; 5 min), or mock treated (ctrl) and collected for lipid extraction at the indicated time points. Cholesterol and cholestenone amounts were determined; n = 8–16. B) HDFs were treated as in A, and wounded with a pipette tip. Cells were fixed at the indicated time points, stained with Alexa568-phalloidin and imaged. Scale bars 20 µm. C) Cell migration area was quantified at 22 h post treatments; n = 11–15 fields. D) HDFs were incubated with MBCD-cholestenone (MB-CN) complex, collected for lipid analysis and cholesterol and cholestenone amounts were determined; n = 6–12. E) HDFs were treated with MB-CN complex as in D, wounded with a pipette tip and incubated in serum-free buffer for 22 h. Cells were stained with Alexa568-phalloidin and the wound area was imaged.

To assess how persistent the cholesterol manipulations in coase- vs. MBCD-treated HDFs were, we analysed the cellular cholesterol and cholestenone content after the treatments. This showed that cells treated with MBCD recovered their cholesterol content within 3 h **(** **Figure 5A** **)**. In contrast, in coase treated cells cholestenone and the accompanying cholesterol reduction persisted at 3 h as well as at 22 h post oxidation **(** **Figure 5A** **)**.

The long-lasting effects of coase treatment make it a potential tool for analysing its functional consequences in the time scale of hours to days. We have recently reported that cholesterol is transported to the plasma membrane to support cell migration [31]. We therefore compared how coase and mild MBCD treatment causing ∼25% cholesterol depletion affected HDF migration in a wound-healing assay. We found that the migration of coase treated cells was reduced by ∼65% in comparison to MBCD or mock treated cells (**Figure 5B, C**). To rule out the possibility that residual cell associated cholesterol oxidase or putative unspecific oxidation products cause the defective cell migration we exchanged ∼20–30% of cellular cholesterol to cholestenone from MBCD-cholestenone complex (**Figure 5D**). The cholesterol/cholestenone content and migration of cells were then assessed at 22 h. This showed that cholestenone remained in cells (**Figure 5D**) and that cell migration was reduced by up to 90% (**Figure 5E**). This strengthens the argument that cell migration is inhibited by cholestenone rather than by some other coase effect.

### Defective lamellipodial formation in cholestenone containing cells

**Figure 6:**
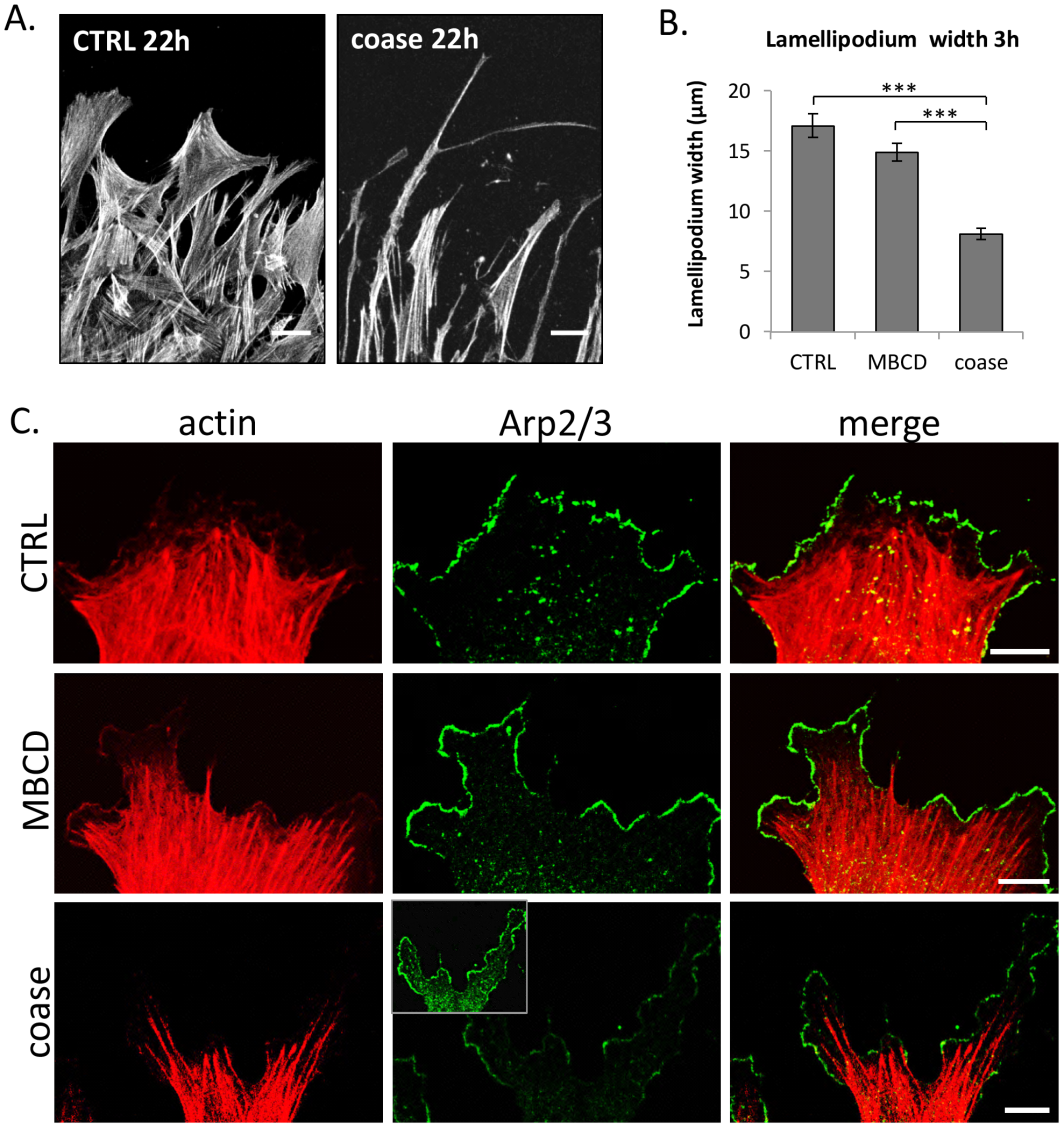
Defective lamellipodial spreading of migrating coase treated cells. A) HDFs were treated, wounded and stained with phalloidin as in Figure 5. B) The width of the lamellipodium was measured from control, MBCD and coase treated cells. Data represent 4 biological replicates per condition; n = 172–228 cells. C) Cells were fixed at 3 h post wounding and stained with fluorescent phalloidin, and anti-Arp2/3 complex component ARPC2 antibody, followed by secondary fluorescent antibody and imaging by confocal microscopy. Please note comparable image acquisition and display in Arp2/3 panels; inset with increased brightness demonstrates the largely cytoplasmic staining pattern of Arp2/3 in coase treated cells. Scale bars, 10 µm.

We noted that the coase-treated cells extended long filopodia-like protrusions toward the wounded area (**Figure 6A**). To elucidate some of the early changes accompanying the non-migratory phenotype of coase-treated cells, we analysed actin in HDFs at 3 h post wounding. Phalloidin staining revealed that in coase treated cells the actin rich protrusions at the leading edge were very narrow and spindle shaped, whereas control and MBCD-treated cells exhibited prominent well spread lamellae (**Figure 6C**). At this time point, the average width of the lamellipodium in coase-treated cells was reduced by over 50% (**Figure 6B**). Lamellipodium spreading depends on the actin branch point complex Arp2/3 [32]. We found that the Arp2/3 complex component ARPC2 was efficiently recruited to the lamellipodia of control and MBCD-treated cells at 3 h post treatment (**Figure 6C**). In contrast, the coase-treated cells displayed a prominent cytosolic ARPC2 staining, and reduced immunostaining at the leading edge (**Figure 6C**). These results suggest that actin dynamics is aberrantly regulated in the presence of cholestenone. This agrees with earlier reports showing that coase treatment affects the diffusion of membrane lipids and proteins in an actin dependent manner [4], [7].

An interesting question is how precisely cholestenone perturbs actin dynamics. As there is no evidence of direct steroid-actin interaction, it seems likely that cholestenone acts via other membrane lipids, such as phosphoinositides (PIPs). PI(4,5)P_2_ and PI(3,4,5)P_3_ regulate lamellipodia formation through binding to actin interactors, such as WAVE/WASP complex proteins that also regulate the activity of the Arp2/3 complex [33]. Interestingly, PI(3,4,5)P_3_ production is specifically needed for lamellipodial spreading during fibroblast migration [34], suggesting that coase treatment might interfere with PI(3,4,5)P_3_ generation. In the future it will be interesting to address the effects of cholestenone on PIPs and their modifying enzymes, to further elucidate how cholestenone perturbs membrane lipids and proteins.

In summary, this study shows that bacterial cholesterol oxidase treatment results in pronounced changes in cell membrane organization. Cholestenone decreases membrane order, undergoes rapid transbilayer movements, desorbs from the membrane more readily than cholesterol and influences cholesterol flip-flop and efflux. In addition, we demonstrate that cholesterol oxidase treatment results in long-term perturbation in cell motility, a process that is highly dependent on membrane lipid composition. This defect appears to be due to the generated cholestenone that persists in cell membranes, affecting e.g. actin regulatory proteins involved in the formation of cell protrusions. Considering that the effect of cyclodextrin mediated cholesterol reduction is reversed within a few hours, the longer-term effects of cholestenone may be useful in instances where an acute cholesterol manipulation is desired to produce prolonged cellular effects.

## Materials and Methods

### Cells and media

Primary HDFs (AG08498 from Coriell Cell Repositories) were grown in DMEM (Lonza) supplemented with 10% non-heat inactivated fetal bovine serum (FBS) (Gibco), 100 U/ml penicillin and streptomycin, and 2 mM L-glutamine.

### Coase and MBCD treatments

For cholesterol oxidation, cells were incubated in Dulbecco's phosphate buffered saline, calcium, magnesium (Gibco), 10 mM Hepes (pH 7.3), glucose (1 g/l) (DPBS+) containing 10 U/ml coase (Streptomyces sp., Calbiochem) for 1 h at 37°C. For moderate cholesterol depletion, cells were subjected to 0.5 mM MBCD (Sigma) for 5 min at 37°C, and for more extensive cholesterol extraction, to 5 mM MBCD for 1 h at 37°C. For cholesterol/cholestenone exchange, cells were treated with 0.5 mM MBCD-cholestenone complex (MBCD/cholestenone 6∶1) for 15 min at 37°C.

### Biochemical lipid analysis

Lipids were extracted according to Bligh and Dyer [35] and separated on HP-TLC by using hexane/diethyl ether/acetic acid (80∶20∶1) as the running solvent. Lipids were visualised by dipping the TLC plate in 3% CuSO_4_, 8% H_2_PO_4_ and charring. Lipids were identified based on standards, and the signal intensity was quantified using ImageJ software. Cholestenone was the only product detected in cells treated by coase (**Figure S2A**). Radiolabeled lipids were scraped from the TLC plate according to lipid standards directly into Optiphase ‘Hisafe’ 3 scintillation cocktail (Perkin Elmer) and quantified by scintillation counting. No radioactivity above background was detected outside the cholesterol and cholestenone regions, except for a low signal corresponding to cholesteryl esters, suggesting that [^14^C]cholesterol and [^14^C]cholestenone are not catabolized in fibroblasts.

### LD isolation

RAW 264.7 macrophages were grown in DMEM, 10% FBS, 10 mM Hepes (pH 7.3). Two 75 cm^2^ flasks of RAW 264.7 macrophages were loaded with 400 µM oleic acid –BSA complex and labeled with 0.1 µM 4-[^14^C]cholesterol (specific activity, 53.0 mCi/mmol, Perkin Elmer) for 18 h in DMEM, 10% FBS, 10 mM Hepes (pH 7.3). Cells were washed twice with PBS and treated with 10 U/ml coase for 2 h 37°C in DPBS+ or mock treated in buffer only. Cells were collected in PBS, and lysed in 1.7 ml of hypotonic lysis buffer (HLB) [20 mM Tris–HCl, pH 7.4; 1 mM EDTA] including a protease inhibitor mixture (chymostatin, leupeptin, antipain, and pepstatin A; 20 µg/ml each) on ice. The lysate was centrifuged at 4°C, 1000×g for 10 min to remove the nuclei. Then, 1.3 ml of the supernatant was mixed with 0.65 ml of 60% sucrose solution to reach a final concentration of 20% sucrose. The samples were transferred to SW50 tubes, and overlaid with 1.7 ml 5% sucrose in HLB and 1.3 ml HLB. The samples were centrifuged for 2 h at 28 000×g, and the LD fraction was recovered from the top of the gradient by using a tube slicer; six additional fractions were collected and the fractions were immediately used for lipid extraction.

### Preparation of BODIPY-lipid/MBCD complex

Bpy-cholestenone was prepared via a ruthenium-catalyzed Oppenauer-type oxidation [36] of Bpy-cholesterol by the following procedure. A solution of Bpy-cholesterol (3.3 mg, 5.7 µmol) in 2 ml of acetone was purged with nitrogen for 10 min. The commercially available Shvo catalyst (2.0 mg, 1.8 µmol) was quickly added, and the resulting solution was heated at reflux (56°C) with stirring under nitrogen for 4 h. After the reaction mixture was cooled to room temperature, the solvent was removed under reduced pressure. The residue was purified by flash chromatography (hexane/EtOAc 5∶1 to 4∶1) on silica gel to afford 1.3 mg (40% yield) of Bpy-cholestenone and 0.8 mg (24% yield) of BODIPY-cholest-4-en-3,6-di-one, which were eluted in separate fractions. Data for Bpy-cholestenone: ^1^H NMR (500 MHz, CDCl_3_) δ 0.75 (s, 3H), 0.80–1.88 (m, 25H), 1.99–2.09 (m, 2H), 2.24–2.42 (m, 2H), 2.43 (s, 6H), 2.52 (s, 6H), 2.72 (dt, *J* = 2.7, 12.8 Hz, 1H), 3.15 (dt, *J* = 5.7, 12.8 Hz, 1H), 5.73 (s, 1H), 6.05 (s, 2H); ESI-HRMS (M+H−HF)^+^ calcd for C_36_H_49_BFN_2_O^+^ 555.3923, found 555.3925. Data for BODIPY-cholest-4-en-3,6-di-one: ^1^H NMR (500 MHz, CDCl_3_) δ 0.75 (s, 3H), 0.80–1.80 (m, 19H), 1.84–1.95 (m, 3H), 1.99–2.07 (m, 1H), 2.11–2.18 (m, 2H), 2.43 (s, 6H), 2.47–2.49 (m, 1H), 2.52 (s, 6H), 2.67 (dd, *J* = 4.0, 16.0 Hz, 1H), 2.73 (dt, *J* = 3.1, 12.8 Hz, 1H), 3.16 (dt, *J* = 5.6, 12.8 Hz, 1H), 6.05 (s, 2H), 6.17 (s, 1H); ESI-HRMS (M+H)^+^ calcd for C_36_H_48_BF_2_N_2_O_2_^+^ 589.3778, found 589.3774.

Bpy-cholesterol was synthesized as described [37]. 370 mM MBCD solution was added to Bpy-cholesterol and Bpy-cholestenone in a 100∶1 molar ratio. The Bpy-lipids were solubilized by bath sonication for 6×5 min at 37°C, and undissolved Bpy-lipids were removed by centrifugation (15 min, 16 000×g). The complexes were stored at 4°C and used within 2 weeks of preparation.

### Fluorescence imaging

Bpy-cholesterol and Bpy-cholestenone were incorporated into HDFs by incubating the cells for 8 min with 0.18 mM MBCD complexed with the lipid. Cells were washed twice with PBS and immediately imaged with a Leica SP2 confocal microscope (Leica Microsystems) with 63× (NA = 0.90) water dipping objective, in CO_2_ independent medium (Gibco) containing 10% FBS. Fluorescent lipids were extracted and separated by HP-TLC using hexane/ethyl acetate 4∶1 running buffer and visualised by a FLA-900 imager (GE Healthcare) with 488 nm wavelength. Based on HP-TLC, no major catabolic products were generated from either Bpy-cholesterol or Bpy-cholestenone (**Figure S2B**). For LD detection, cells were co-labeled with 5 µM BODIPY558/568-C12 (Life Technologies) and 1 µM Bpy-cholesterol or 1 µM Bpy-cholestenone in DMEM containing 0.2% DMSO for 1 h. Cells were imaged live after a 1 h chase. For immunofluorescence microscopy, cells were fixed at room temperature with 4% paraformaldehyde (PFA), and permeabilized with 0.1% Triton X-100 for 5 min. Cells were stained in 10% FBS in PBS by incubating with primary antibodies at 4°C overnight, then with secondary antibodies for 45 min at 37°C, and finally mounted in Mowiol containing 2.5% 1,4-diazabicyclo[2.2.2]octane. Confocal images were acquired with a Leica TCS SP8 confocal microscope (Leica Microsystems), with 40× (NA = 1.1) water or 60× (NA = 1.3) glycerol objectives. Anti-ARPC2 was purchased from Upstate (p34Arc) (07-277).

### Efflux assays

Bpy-lipids were incorporated into HDFs from a MBCD complex as above. To analyse the efflux of Bpy-lipids, cells were washed twice with PBS and immediately lysed in 1% NP-40, or incubated for 4 h in DMEM, 10% FBS, washed twice, and lysed. Bpy-fluorescence was measured using a Victor microplate reader (Perkin Elmer). To analyse the efflux of radiolabeled lipids, HDFs were labeled with 0.2 µCi/ml 4-[^14^C]cholesterol for 42 h in DMEM, 10% FBS. Cells were washed with PBS to remove excess label, and treated with coase and MBCD as described above. Cells were then washed and incubated in DPBS+ containing 10 µg/ml ApoA-I or 0.2% BSA for 4 h. Cells and media were collected and lipids were extracted and analysed by HP-TLC.

### Wound-healing assay

Confluent HDF monolayers were scratched with a pipette tip. Cells were incubated in DPBS+, fixed at the indicated time points, stained with Alexa568-phalloidin (Molecular probes) and imaged with an AX70 epifluorescence microscope (Olympus) equipped with an Olympus DP71 CCD camera using a 4× objective. Images were analysed with ImageJ software by thresholding and automatically measuring the wound area. The migration area was calculated as the difference between the wound area at 0 h and 22 h post wounding.

### Generalized polarization analysis

GP is described as: (*I_400–460_−I_470–530_*)/(*I_400–460_+I_470–530_*). Cells were labeled with 5 µM Laurdan in serum-free DMEM for 45 min at 37°C, washed, and fixed in 4% PFA. Mounted coverslips were imaged with a Leica TCS SP8 confocal microscope, with 63× (NA = 1.3) glycerol objective, by two-photon imaging. Laurdan was excited at 800 nm and emission was recorded at 400–460 and 470–530 nm. The images were analysed with ImageJ software by using a GP analysis macro provided in [38] and corrected for G factor obtained from measuring Laurdan emission in pure DMSO [38]. The resulting images were generated as a merge of Laurdan fluorescence intensity in ordered channel and GP value in the corresponding pixel. Data were normalized to an average GP value of control samples from different experiments.

### Statistical analysis

Student's t-test was used for statistical analysis. Data are reported as mean +/− standard error of mean (SEM).

### Atomistic simulations and analysis of simulation data

We studied two raft-like lipid bilayers through unbiased MD simulations (**Table 3**). One of them was composed of 256 POPC, 128 PSM, and 128 cholesterol (chol) molecules (POPC/PSM/cholesterol/cholestenone; molar ratio of 2∶1∶1∶0) (system I_MD_). In the other, part of cholesterol molecules were replaced with cholestenone, and this system contained 256 POPC, 128 PSM, 102 cholesterol, and 26 cholestenone (cnone) molecules (POPC/PSM/cholesterol/cholestenone; molar ratio of 2∶1∶0.8∶0.2) (system II_MD_). Both bilayers were hydrated with approximately 30000 water molecules. The initial membrane structures were obtained from our previous study [39]. To parameterize the interactions in the systems, for lipid molecules we used the all-atom optimized potentials for liquid simulation (OPLS) force field [40], following our previous works [41], [42]. This set of charges was derived in compliance with the OPLS methodology. For water, we employed the TIP3P model, which is compatible with the OPLS parameterization [43].

**Table 3:** Lipid compositions for all the bilayers^a^ used in the present work.

System	POPC	PSM	Cholesterol	Cholestenone	Phase	Area per lipid^b^ (Å^2^)
I_MD_	256	128	128	0	L_o_	59.31±0.57
II_MD_	256	128	102	26	L_o_	59.20±0.53
I_FE_	64	32	32	0	L_o_	59.01±0.91
II_FE_	64	32	24	8	L_o_	58.96±1.01
III_FE_	64	32	0	32	L_o_	57.95±1.37
IV_FE_	127	0	1	0	L_d_	68.75±1.23
V_FE_	127	0	0	1	L_d_	68.11±1.33

a The subscripts “MD” refer to the systems studied only by regular (unbiased) molecular dynamics simulations, thus they were not used in free energy calculations. The subscripts “FE” refer to the systems used for free energy calculations. L_o_ and L_d_ stand for liquid-ordered and liquid-disordered phases, respectively.

b Area per lipid calculated by dividing the time averaged total area of each simulation system by the number of lipids per leaflet.

The numbers of each lipid in a given system, the phase, and the area per lipid are indicated.

Simulations were performed with GROMACS software [44], using the GROMACS 4.6.5 package. All MD simulations were 200 ns long, and the first 20 ns were considered as an equilibration period. Periodic boundary conditions with the usual minimum image convention were used in all three directions. The LINCS algorithm [45] was used to preserve hydrogen covalent bond lengths. The time step was set to 2 fs for numerical integration. Simulations were carried out under isothermal-isobaric (310 K, 1 bar) conditions. The temperature and pressure of the systems were controlled by the v-rescale thermostat and the Parrinello-Rahman barostat [46], [47] with time constants of 0.1 ps and 1 ps, respectively. The temperatures of the solute and the solvent were controlled independently. For pressure, we used a semi-isotropic control. The Lennard-Jones interactions were cut off at 1.0 nm, and for the electrostatic interactions we employed the particle mesh Ewald method [44] with a real space cutoff of 1.0 nm, β-spline interpolation (order of 6), and direct sum tolerance of 10^−6^.

In free energy (FE) calculations with the umbrella sampling technique [19] we used the same protocol as in unbiased MD simulations. However, because of a large computational cost of FE calculations, the system size was reduced to one-fourth of that used in **Table 3**. The bilayers studied for FE computations were: (i) system I_FE_ – composed of 64 POPC, 32 PSM, and 32 cholesterol molecules (POPC∶PSM∶cholesterol∶cholestenone 2∶1∶1∶0), (ii) system II_FE_ – composed of 64 POPC, 32 PSM, 24 cholesterol, and 8 cholestenone lipids (POPC∶PSM∶cholesterol∶cholestenone; molar ratio of 2∶1∶0.75∶0.25), and (iii) system III_FE_ – in this bilayer cholesterol was completely replaced by cholestenone and it contained 64 POPC, 32 PSM, and 32 cholestenone molecules (POPC∶PSM∶cholesterol∶cholestenone; molar ratio of 2∶1∶0∶1). In addition to liquid-ordered (L_o_) membranes, we also simulated two liquid-disordered (L_d_) bilayers containing 127 POPC molecules with either one cholesterol (system IV_FE_) or one cholestenone (system V_FE_) present in one of the two leaflets.

Before the FE calculations commenced, all bilayers were simulated for 100 ns, of which the first 20 ns was considered as equilibration and the rest of these data were used for analysis. We then calculated the free energies of translocation (flip-flop) of steroid molecules from their equilibrium position to the bilayer center and further to the other leaflet, as well as the FE of steroid desorption from a bilayer to the water phase. In these calculations, we restrained the hydroxyl group of cholesterol and the ketone oxygen of cholestenone together with three adjacent ring carbon atoms for each steroid with respect to the center of mass of the membrane, applying a harmonic restraint with a force constant of 750 kJ mol^−1^ nm^−2^. In each adjacent window, cholesterol/cholestenone was moved by 0.1 nm in the direction normal to the bilayer, with a total of 41 simulations to sample the entire reaction coordinate. The windows were run for 50–100 ns. The potential of mean force was calculated by the weighted histogram analysis method (WHAM) [48], using the g_wham tool implemented in GROMACS. The statistical errors were estimated with bootstrap analysis [48].

The conformational ordering of lipid acyl chains was characterized by the order parameter S_CD_ = ½<3 cos^2^θ−1>, where θ is the angle between the C–H bond vector and the membrane normal. The angular brackets indicate averaging over time and lipids. The largest value of 0.5 corresponds to full conformation order (all-trans), while values close to zero describe a situation with no conformational order (disordered acyl chains). The overall shape of our order parameter profile, including the dip at smaller carbon numbers, is in qualitative agreement with the previous NMR-derived order parameter profile for POPC lipid [49].

The presence of hydrogen bonds was evaluated based on distance and angle criteria: the donor- acceptor distance had to be less than or equal to 0.325 nm, and the angle between the vector linking a donor and an acceptor, and the vector describing the chemical bond between hydrogen and a donor had to be less than or equal to 35°. The existence of charge pairs was determined using the distance cut-off of 0.4 nm. The geometric criterion used in hydrogen bond and charge-pair estimation was derived from our previous work [50].

The rates of cholesterol and cholestenone flip-flop were calculated using the equation *k_f_* = *k_d_*×exp (−ΔG_center_/RT), where ΔG_center_ is the difference in free energy between the bilayer center (Z_center_) and the equilibrium position Z_eq_ that is the position of the free energy minimum. Then k_f_ is the rate constant to move from Z_eq_ to Z_center_, and k_d_ is the rate constant with which a molecule returns from Z_center_ to Z_eq_ in terms of *k_d_* = 1/*t_d_*. Here the time t_d_ is the estimated time-range for steroid molecules to move from Z_center_ to Z_eq_. This can be estimated by releasing the harmonic restraint on a steroid at Z_center_, and then computing the time that it takes to return back to Z_eq_. Instead of attempting a more rigorous estimation, a time range of 1–80 ns was used for cholesterol (considering the previous report on cholesterol flip-flop in different bilayer membranes, including raft-mimicking environments [20]). The same value was used for cholesterol in all bilayers. For cholestenone, a time range of 1–40 ns was used for all bilayers (the time range was based on present findings and an earlier report by Róg et al. [51]). Since a complete flip-flop event involves the rate to move from bilayer equilibrium to the center (k_f_) and then from the center to the opposite leaflet (k_d_), the rate constant of flip-flop is expressed as *k_flip_* = ½×1/[*k_f_^−1^*+*k_d_^−1^*]≈*k_f_*/2.

## Supporting Information

Figure S1 **The mean-squared displacement (MSD) of palmitoyl-sphingomyelin (PSM) in the membrane plane.** The lipid compositions of the studied systems differed with respect to cholesterol/cholestenone as indicated. The black line represents the lateral displacement of PSM in the raft-like bilayer containing 25% cholesterol, while the red line represents the corresponding MSD data when all membrane cholesterol was replaced by cholestenone. In the calculation of the MSD, the first 20 ns were removed from the simulation trajectory. Mean-squared displacement (MSD) of single-lipid motion for PSM was calculated: MSD(*t*) = <|*r_i_* (*t*+*τ*)−*r_i_* (*τ*)|^2^>*_i,τ_*], where *r_i_*(*t*) is the position of lipid *i* at time *t*, and < >*_i,t_* stands for averaging over the lipids *i* = 1,2,…,*N* and the times *τ*. Then the lateral diffusion coefficient for the given lipid type is D*_L_* = lim_τ→∞_[1/(4*t*)<|*r_i_* (*t*+*τ*)−*r_i_* (*τ*)|^2^>*_i,τ_*]. The center of mass motion of individual leaflets was removed before calculation as monolayers can drift during the simulation.
(TIF)

Figure S2 **Chromatographic analysis of cholesterol, cholestenone and their fluorescent analogs extracted from HDFs.** A. HDFs were treated with coase (10 U/ml; 1 h at 37°C). Cells were collected after 22 h chase and the lipids were extracted and analysed by HP-TLC. Standards: CHOL = cholesterol, CNONE = cholestenone, TG = triglycerides, CE = cholesteryl esters. B. HDFs were pulse labeled for 30 min with 1 µM Bpy-cholesterol (Bpy-chol) or Bpy-cholestenone (Bpy-cnone). Cells were collected immediately after labeling (0 h) or after 4 h chase, and the lipids were extracted and analysed by HP-TLC.
(TIF)
