# Supplementary figures and images for: Enzymatic Oxidation of Cholesterol: Properties and Functional Effects of Cholestenone in Cell Membranes
Source: PLoS One. 2014 Aug 26;9(8):e103743. doi: 10.1371/journal.pone.0103743 (PMC4144813; doi:10.1371/journal.pone.0103743)

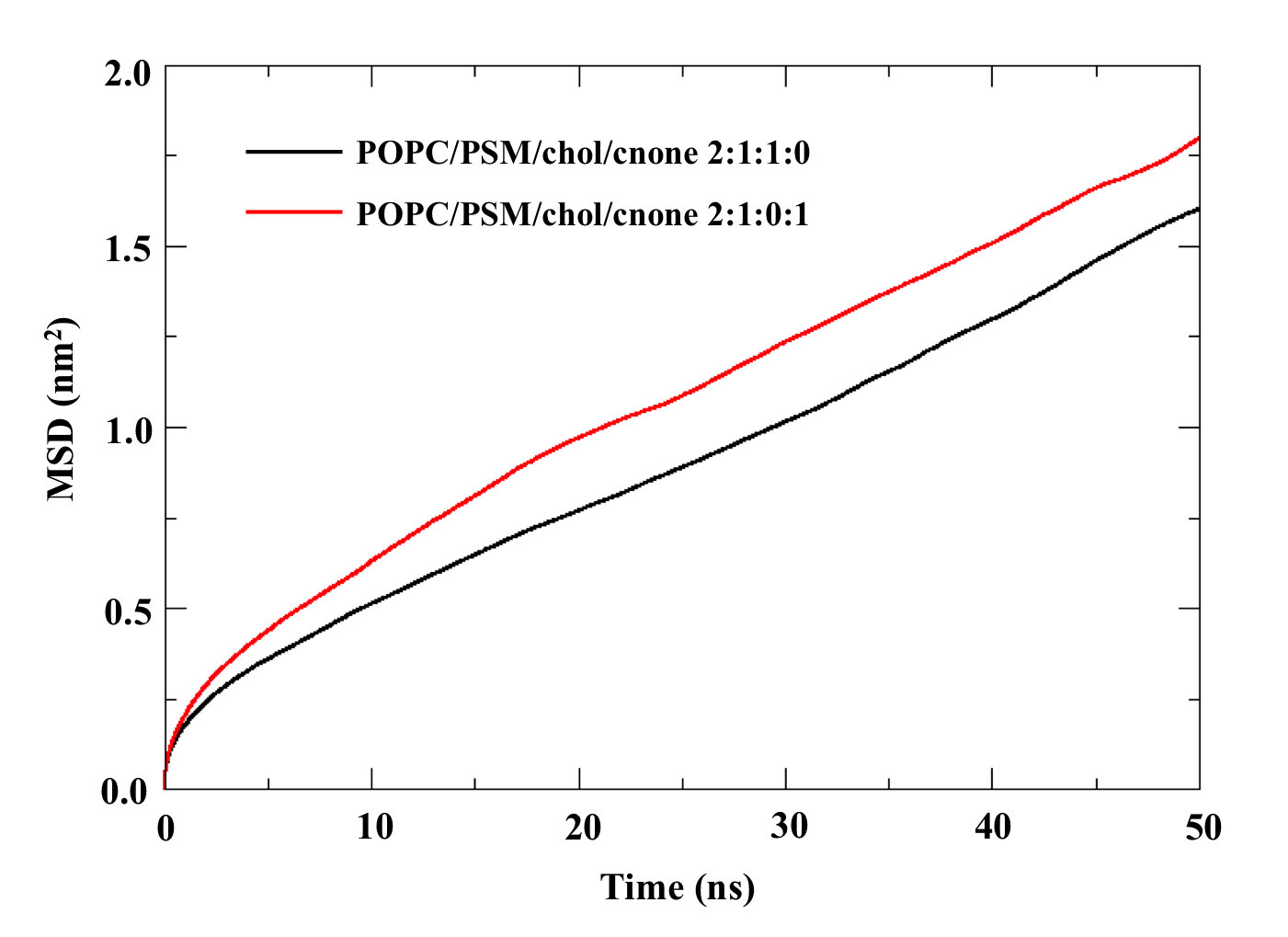

Supplement: Figure S1 — The mean-squared displacement (MSD) of palmitoyl-sphingomyelin (PSM) in the membrane plane. The lipid compositions of the studied systems differed with respect to cholesterol/cholestenone as indicated. The black line represents the lateral displacement of PSM in the raft-like bilayer containing 25% cholesterol, while the red line represents the corresponding MSD data when all membrane cholesterol was replaced by cholestenone. In the calculation of the MSD, the first 20 ns were removed from the simulation trajectory. Mean-squared displacement (MSD) of single-lipid motion for PSM was calculated: MSD(t) = <|ri (t+τ)−ri (τ)|2>i,τ], where ri(t) is the position of lipid i at time t, and < >i,t stands for averaging over the lipids i = 1,2,…,N and the times τ. Then the lateral diffusion coefficient for the given lipid type is DL = limτ→∞[1/(4t)<|ri (t+τ)−ri (τ)|2>i,τ]. The center of mass motion of individual leaflets was removed before calculation as monolayers can drift during the simulation. (TIF) [file pone.0103743.s001.tif]

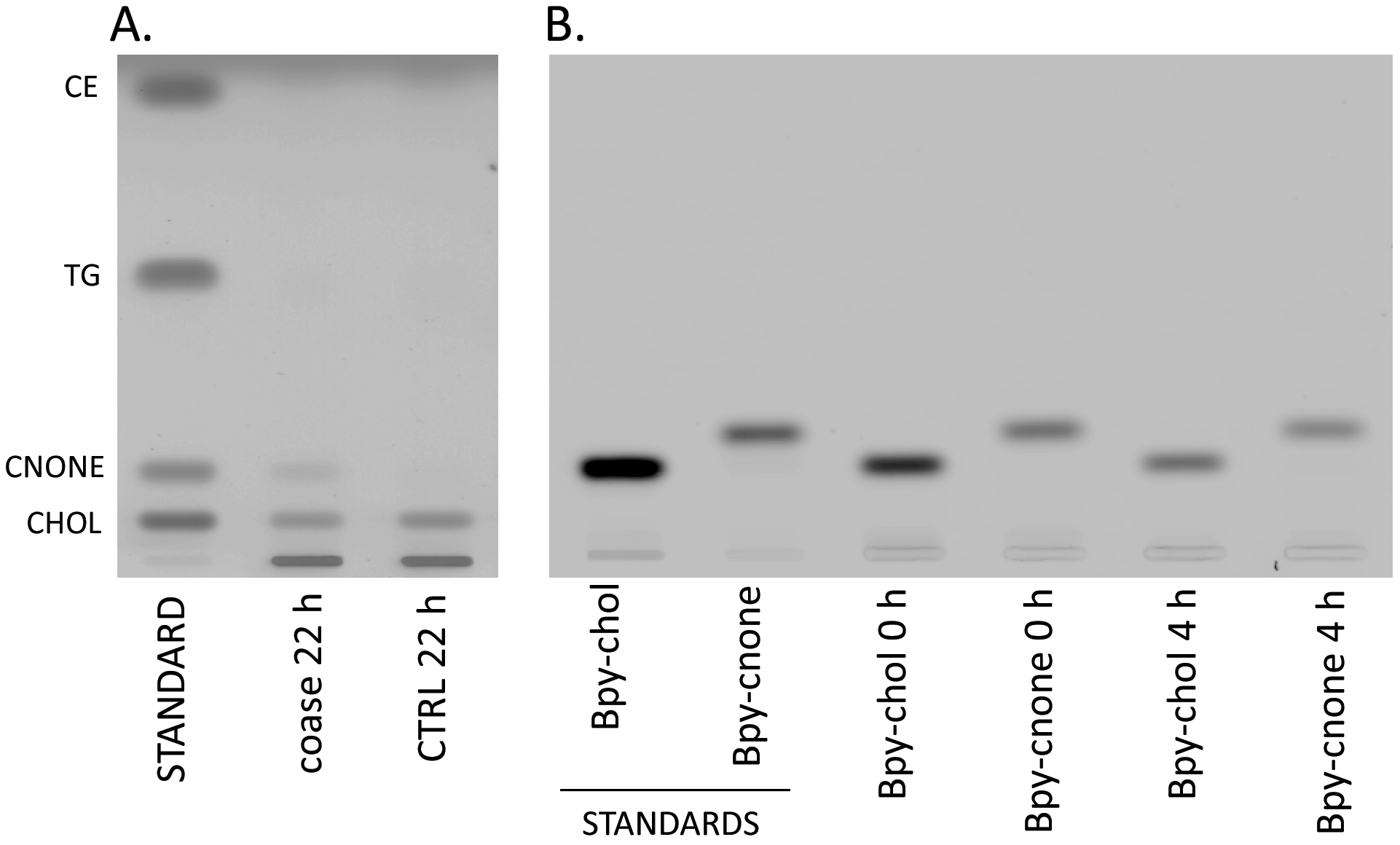

Supplement: Figure S2 — Chromatographic analysis of cholesterol, cholestenone and their fluorescent analogs extracted from HDFs. A. HDFs were treated with coase (10 U/ml; 1 h at 37°C). Cells were collected after 22 h chase and the lipids were extracted and analysed by HP-TLC. Standards: CHOL = cholesterol, CNONE = cholestenone, TG = triglycerides, CE = cholesteryl esters. B. HDFs were pulse labeled for 30 min with 1 µM Bpy-cholesterol (Bpy-chol) or Bpy-cholestenone (Bpy-cnone). Cells were collected immediately after labeling (0 h) or after 4 h chase, and the lipids were extracted and analysed by HP-TLC. (TIF) [file pone.0103743.s002.tif]
